# The construction of a decision tool to analyse local demand and local supply for GP care using a synthetic estimation model

**DOI:** 10.1186/1478-4491-11-55

**Published:** 2013-10-27

**Authors:** Willemijn A de Graaf-Ruizendaal, Dinny H de Bakker

**Affiliations:** 1 Department of Primary Care Organization, NIVEL: Netherlands Institute for Health Service Research, PO Box 1568, 3500 BN Utrecht, The Netherlands; 2 Department for Social and Behavioural Science, Tranzo Tilburg University, PO Box 90153, 5000 LE Tilburg, The Netherlands

**Keywords:** Health workforce planning, Local population demand, Synthetic estimation method, General practitioner care, Spatial microsimulation model, Decision tool

## Abstract

**Background:**

This study addresses the growing academic and policy interest in the appropriate provision of local healthcare services to the healthcare needs of local populations to increase health status and decrease healthcare costs. However, for most local areas information on the demand for primary care and supply is missing. The research goal is to examine the construction of a decision tool which enables healthcare planners to analyse local supply and demand in order to arrive at a better match.

**Methods:**

National sample-based medical record data of general practitioners (GPs) were used to predict the local demand for GP care based on local populations using a synthetic estimation technique. Next, the surplus or deficit in local GP supply were calculated using the national GP registry. Subsequently, a dynamic internet tool was built to present demand, supply and the confrontation between supply and demand regarding GP care for local areas and their surroundings in the Netherlands.

**Results:**

Regression analysis showed a significant relationship between sociodemographic predictors of postcode areas and GP consultation time (*F* [14, 269,467] = 2,852.24; *P* <0.001). The statistical model could estimate GP consultation time for every postcode area with >1,000 inhabitants in the Netherlands covering 97% of the total population. Confronting these estimated demand figures with the actual GP supply resulted in the average GP workload and the number of full-time equivalent (FTE) GP too much/too few for local areas to cover the demand for GP care. An estimated shortage of one FTE GP or more was prevalent in about 19% of the postcode areas with >1,000 inhabitants if the surrounding postcode areas were taken into consideration. Underserved areas were mainly found in rural regions.

**Conclusions:**

The constructed decision tool is freely accessible on the Internet and can be used as a starting point in the discussion on primary care service provision in local communities and it can make a considerable contribution to a primary care system which provides care when and where people need it.

## Introduction

### Responsive primary care

There is a growing academic and policy interest in the appropriate provision of primary healthcare services to the population of local areas to increase health status and decrease healthcare costs [1-3]. Governments and healthcare organisations aim for primary care services that are demand-driven, are easily accessible, locally available and established in accordance with the health criteria of the local population [1,3,4]. However, there are great disparities in the health care use of different sociodemographic and socioeconomic groups [5-7]. Therefore, it is a great challenge to match primary healthcare services to the healthcare needs of the local population. Local information on healthcare needs is necessary to gain more insight into these disparities in order to arrive at a better match between demand and supply.

Unfortunately, it is impossible to acquire local health-related data for every local area, and there are several reasons for this. First, most national health surveys are not designed to generate estimates for small areas; national survey data either do not contain respondents for every small area or the sample size is too small to generate valid estimates [8]. Second, local health surveys are costly and, as a result, they are not routinely updated [9,10]. Third, if local health data are available for some local areas, they are often distributed over fragmented data sources, which makes it difficult to combine and interpret them [11].

### Spatial microsimulation models

To assist organisations and healthcare providers in the supply of local health-related data, spatial microsimulation models can be used. Spatial microsimulation models have a long history in economics and are increasingly used in epidemiology as an alternative to local health surveys [12]. In short, such models construct large synthetic micro data at the small area level on the attributes of individuals or households by combining different sources of information to 'estimate geographical distributions of variables which were previously unknown’ [13], p 1128. There are various types of spatial microsimulation models, varying from models which only construct micro datasets to models which use the constructed micro dataset to build future micro datasets and consider future policy changes [14].

Regarding healthcare issues, micro datasets have been generated for issues such as obesity, mental disorder, access to general practitioner (GP) services and lifestyle behaviour such as smoking and alcohol consumption [9,13,15,16]. Datasets of local health-related data can be used to identify local areas where, for example, the number of people smoking is higher or lower than the national average [9]. These local data could assist policymakers in their decisions regarding the implementation of interventions.

However, for planning purposes it would be more effective if a model incorporated not only the expected demand for care but also the spatial distribution of health services, and thus identified potentially underserved or overserved areas. With this in mind, Morrissey et al. (2008) estimated GP visits in a rural district of Ireland, using a spatial microsimulation model [16]. They assessed whether the spatial distribution of GP services matched the demand at a local level, and they concluded that the demand for GP care was much higher in rural areas than urban areas. However, surprisingly, the accessibility of GP care services was the lowest in these rural areas [16].

In the present study, the work of Morrissey et al. (2008) was expanded [16]. It was investigated to what extent a spatial microsimulation model can be developed and expanded into a dynamic Internet decision tool which can be used to fine-tune the provision of primary care to the demand of the local population for all the local areas in the Netherlands. Not only were underserved or overserved areas identified, but the deficits and surpluses in the number of physicians for the specific areas were also calculated. This article describes how the model was built, what data were used and which method was applied. Moreover, the results of the model are presented and the possible consequences for health policy are discussed. The model generates data regarding almost all primary care disciplines, however, this article focuses on the description of the method and the results in general practice care.

## Methods

### Design

As discussed above, local information on the demand of primary care is often missing. One possible solution is to calculate synthetic estimates of local health demand figures by means of a spatial microsimulation model that uses a synthetic estimation technique. This general technique produces health estimates for local areas for which health data are unknown by using health data from other local areas using a model-based approach [8]. For this technique two datasets are necessary: a national census dataset which includes sociodemographic characteristics of local area, and a national sample-based dataset which includes medical record of GPs for a number of local areas. A synthetic estimation technique was used to estimate local demand for GP care. These estimations were subsequently compared to actual GP supply from the national GP registry to assess the match between supply and demand for local areas and their surroundings.

### Data collection

Sample based medical record data of GPs from 2008 were obtained from the National Information Network of General Practice (LINH) from the Netherlands [17]. This network is a dynamic pool of practices, geographically well-distributed across the Netherlands, with yearly changes in composition. The data used contain approximately 350,000 patients from 85 general practices. Patients listed in the LINH practices are representative of the Dutch population regarding gender and age. The LINH database contains frequency of GP contacts, gender, age and the postcode of each patient registered by GPs. Of the 85 general practices, 13 were excluded because of incomplete data.

National census data were obtained from *Statistics Netherlands* by postcode [18]. For the present study, postcode area was chosen as geographical unit, because postcode area comes closest to the neighbourhood at which primary care services operate. The average population size of a postcode area is 5,771 inhabitants. Data were collected regarding the total population, the numbers of male and female inhabitants in age categories, the number of one-person households, the number of non-Western immigrants: at least one parent is born in Africa, Latin America or Asia, the number of low-income households: households with a purchasing power of < €9,250 a year, and the degree of urbanisation of the area divided into five categories from rural (<500 addresses per km^2^) to very highly urbanized (>2,500 addresses per km^2^) [18]. These area characteristics were selected as predictors because they are known to be important determinants of healthcare use [19]. For instance, women visit their GP more often than men and older people also have a higher GP contact rate [20], as do non-Western immigrants [21] and people with a low income [22]. In addition, people living in rural areas make use of healthcare services more frequently [23]. Other important determinants, such as education, are not available by postcode. The area characteristics were linked to patients by patient’s postcode.

Information on GP supply in the Netherlands was obtained from the national GP registry for the year 2009 [24]. The GP registry contains characteristics for every GP and GP practice in the Netherlands. The number of GPs, the number of full-time equivalents (FTEs) and the postcodes of the general practices were extracted from this database.

### Statistical analysis

**Figure 1:**
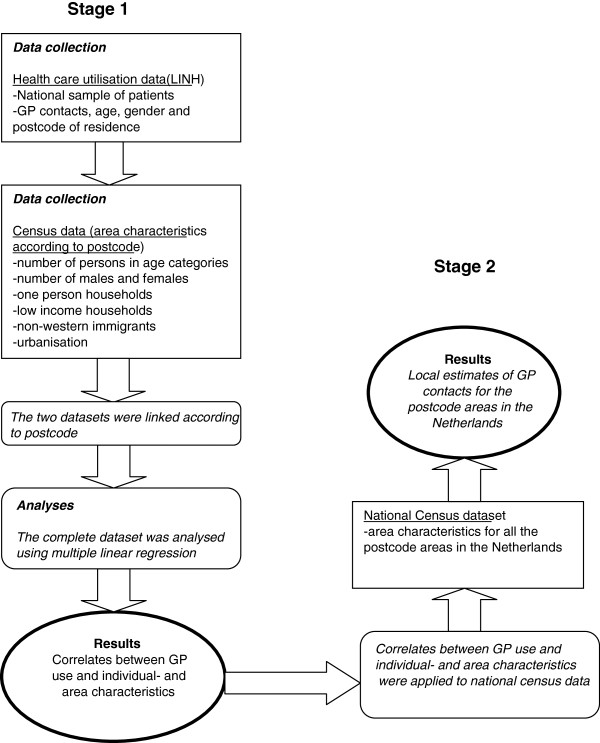
Flow diagram of the methodological approach.

To obtain a spatial micro dataset regarding the estimated demand for care, a synthetic estimation technique was used consisting of two main stages (Figure 1). The first stage involved generating a statistical model which represents the relationship between the demand for GP care and the sociodemographic predictors. GP registration data on patient level were linked to national census data by postcode. In the second stage, the statistical model was applied to national census data in order to estimate the demand for GP care for every postcode area.

Subsequently, multiple linear regression analysis was conducted between the number of contacts with the GP per listed patient and dummies for 'patients gender and age’ (female = 1, male = 0; 0–4 years old, 5–14 years old, 15–24 years old, 25–39 years old = reference category, 40–64 years old, 65–74 years old and 75 years and older), 'proportion one-person households’ , 'proportion low-income households’ , 'proportion non-Western immigrants’ , and dummies for 'urbanisation’ of the area (reference category = rural). The annual number of GP contacts was converted into GP consultation time by multiplying it by 10, because an average GP contact takes about 10 min in the Netherlands [25].

**Figure 2:**
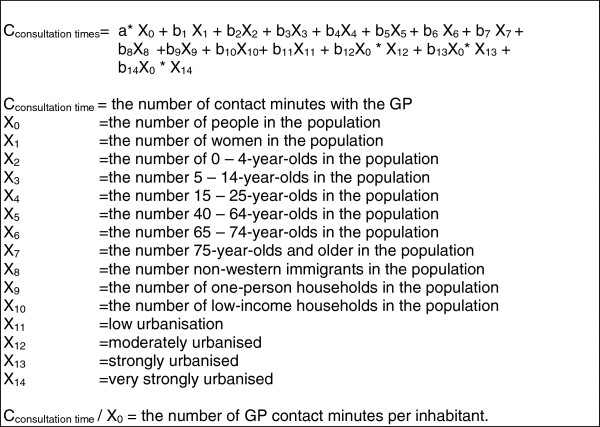
Mathematical model for the expected GP consultation time in minutes per inhabitant.

Next, the coefficients from the multiple linear regression for the different predictors were multiplied by the number of these predictors in the area to estimate GP consultation time for all the postcode areas in the Netherlands (*n* = 4,033; Figure 2). No results are presented for the 1,260 postcode areas with <1,000 inhabitants. Estimations based on <1,000 inhabitants are not considered reliable. The included postcode areas still covered 97% of the total population. The analyses were performed with STATA 10.0 [26].

### Confronting supply and demand

Two parameters were computed as indicators for the match between demand and supply:

1. The expected consultation time per available GP; this indicates the amount of work for the GPs in the postcode area given the local population.

2. The number of FTE GP too much/too few to reach the national average of 7,743 contacts per FTE GP per year. This is an indicator for under- and oversupply. According to the norm, the average FTE GP in the Netherlands has approximately 2,350 patients [24] and the average patient has a GP consultation time of 31.8 min per year [17]. This results in a standard workload for an FTE GP of 74,730 min consultation time.

The two parameters were computed at the level of the postcode area itself and at the level of the postcode area including surroundings. This has been done because not everyone visits the GP in his own postcode area. Therefore, undersupply in the postcode area can be compensated by oversupply in the surrounding area. For this reason, the surrounding postcode areas situated at 3 km or less by road were also analysed for the supply and demand of GP care. In 2004, the mean distance from a patient to his own GP practice was 2.7 km in the Netherlands [27]. This number was rounded up to 3 km because this distance was not measured from the patients’ actual addresses but from the centre of their postcode area. The analysis was conducted with Mapinfo Professional. In the Netherlands, general practice is the formal point of entry into the health care system and GPs function as gatekeepers; specialist and hospital care can only be accessed by referral from a GP. In the Netherlands, GP care operates at a neighbourhood level. All residents are registered with a GP practice usually closest to the residence or on a very small distance. The mean distance to a GP is 2.7 kilometres [27]. The mean number of inhabitants per FTE GP is 2,350. The Dutch government does not intervene actively to realize this standard.

## Results

The results of the multiple regression analysis are presented in Table 1. The regression analysis showed a significant relationship between the predictors of the model and GP consultation time (*F* [14, 269,467] = 2,852.24; *P* <0.001). The model explained 12.9% of the variation in the dependent variable. The results revealed that 11 variables were significant predictors of GP consultation time. The strongest predictors of the number of GP contact minutes were '75 years and older’ (*B* = 55.1, *P* <0.001) and '65-74 years old’ (*B* = 25.5, *P* <0.001).

**Table 1:** Regression coefficients for annual GP consultation time in minutes

	**b**	**Lower bound**	**Upper bound**
		**95% CI**	**95% CI**
Constant^a^	15.33892^b^	14.42749	16.25035
Female	11.92347^b^	11.59783	12.2491
0-4 years	0.411849	-0.354851	1.178549
5-14 years	-9.006723^b^	-9.596515	-8.416931
15-24 years	-3.599869^b^	-4.183009	-3.016729
40-64 years	7.796694^b^	7.345136	8.248252
65-74 years	25.50999^b^	24.814	26.20598
75 years and older	55.09777^b^	54.35037	55.84517
Proportion non-Western immigrants	9.313317^b^	7.663707	10.96293
Proportion one-person households	-2.330715	-4.831568	0.170138
Proportion persons in low-income households	18.94194^c^	15.96651	21.91738
Low urbanisation	-1.031837^b^	-1.567949	-0.495726
Moderately urbanised	0.153963	-0.426263	0.734188
Strongly urbanised	-0.579397^c^	-1.158326	-0.000468
Very strongly urbanised	-3.874945^b^	-4.837014	-2.912876

^a^Constant = male, 25–39 years, no non-Western immigrants, more person household, no low income, no urbanisation.

^b^*P* <0.01.

^c^*P* <0.05. CI, Confidence intervals; r^2^ = 12.9%.

### GP demand

The results of the mathematical model (Figure 2) showed an average GP consultation time per postcode area of *χ* = 183,650 (SD = 122,944) and per inhabitant per year of *χ* = 31.9 (SD = 3.6). The postcode area with the lowest expected GP consultation time (*χ* = 21.6) had a low percentage of low-income households, a low percentage of one-person households, a low percentage of people older than 65 years and a low level of urbanisation.

### GP supply rates

The mean number of FTE GPs was highest in strongly urbanised postcode areas (*χ* = 3.8; SD = 3.0) and lowest in rural postcode areas (*χ* = 1.8; SD = 1.9).

### Confronting supply and demand

The comparison between expected GP consultation time based on the sociodemographic profile of the postcode areas and actual GP supply revealed a shortage of GP supply for 54% of the postcode areas, if an average workload of 74,730 contact min per FTE GP was assumed. The total shortage for these areas was 1,653 FTE GP. A GP shortage >1 FTE was prevalent for about 20% of the postcode areas with a total of 4 million inhabitants.

The results of the surrounding analysis showed a shortage of GP supply for 46% of the areas, so for 8% of the postcode areas there is compensation. A shortage >1 FTE was indicated in 19.0% of the postcode areas. Together, these areas had a total shortage of 1,417 FTE GPs for >3 million people.

The expected workload per FTE GP in the Netherlands was 76,360 contact min a year (SD = 47,869). When the surrounding areas were taken into consideration the mean expected workload per FTE GP was 75,617 contact min per year (SD = 40,754). Table 2 relates different classes of GP workload to the proportion of the Dutch population and shows a large variation in the workload of GPs. The majority of the Dutch population lives in a postcode area with a workload of 50,000-100,000 GP contact min. However, the surrounding analysis showed that, respectively, 8.6%, 3.0% and 0.4% of the Dutch population live in a postcode area with a higher workload than the norm workload. Moreover, 4.9% of the Dutch population have no GP in their postcode area and surrounding area.

**Table 2:** Distribution of Dutch postcode areas and population over classes of expected workload

	**For postcode areas with >1,000 inhabitants (*n*** **= 2,773)**		**For postcode areas (*n*** **= 2,773) and their surrounding areas**	
**Workload: Annual GP consultation time (min)**	**Postcode areas (%)**	**Inhabitants of the total Dutch population (%)**	**Postcode areas (%)**	**Inhabitants of the total Dutch population (%)**
8,000-50,000	21.0	17.9	13.2	8.6
50,000-100,000^a^	40.0	47.1	62.6	71.5
100,000-150,000	9.4	12.9	7.2	8.6
150,000-300,000	4.4	5.7	2.9	3.0
300,000-500,000	0.4	0.7	0.4	0.4
No GP	24.8	12.7	13.7	4.9

^a^The norm workload for a Dutch GP is 74,730 min per year.

The average shortage/surplus in FTE GP per postcode area including surroundings was 0.67 (SD = 3.4). So, overall there was no shortage in FTE GP supply when GP supply was confronted with the estimated GP consultation time based on the sociodemographic composition of the postcode areas. However, GP supply was unequally dispersed over the expected demand for GP care. Table 3 shows the percentage of the shortage or surplus in FTE GPs related to the number of FTE GPs needed to cover the expected demand in postcode areas and their surroundings. The resulting shortage or surplus is represented for areas with different population sizes. The mean percentage surplus in FTE is 0.23%. Areas with the fewest inhabitants showed the largest percentage of shortage in FTEs. Most of these areas were rural areas. In contrast, areas with the highest numbers of inhabitants had the largest percentage of surplus in FTE GPs. This indicated that urban areas probably compensate the shortage in rural areas.

**Table 3:** **The percentage shortage/surplus in FTE GPs for different area sizes including the surrounding areas (*n*** **= 2,773)**

**Residents class**	**Mean FTE GP needed based on the expected demand for GP care**	**Actual mean FTE GP supply**	Mean % shortage/surplus in FTE GP based on needed GP care*	**Postcode (*n*)**
1,000-2,500	0.70	0.60	-18.9	553
2,500-5,000	1.58	1.67	5.89	372
5,000-7,500	2.66	2.68	-0.23	251
7,500-10,000	3.84	3.87	0.19	199
10,000-15,000	5.38	5.69	5.19	289
15,000-20,000	7.54	7.91	4.93	200
20,000-30,000	10.49	10.75	2.93	340
>30,000	25.74	28.59	9.59	569

Mean percentage shortage/surplus in FTE GP = ((FTE GP - needed FTE GPs)*100)/FTE GPs needed for every postcode area including the surrounding areas.

## Discussion

The distribution of GPs is usually based on the number of inhabitants in an area, on the attractiveness of the area for GPs regarding work opportunities or personal factors. However, this may lead to underserved or overserved areas [28], while governments aim for primary care services which are locally available and accessible. This study presents the method and the results of a decision tool which not only makes it possible to analyse the estimated demand and the supply of GP care, but also the confrontation between supply and demand for GP care for local areas in the Netherlands. The results showed that the constructed model could estimate GP consultation time for every area with >1,000 inhabitants in the Netherlands covering 97% of the total population. Confronting these estimated figures with the actual GP supply resulted in the average GP workload and the number of FTE GP too much/too few for local areas to cover the demand for GP care. If the surrounding postcode areas were taken into consideration, 19% of the areas had a shortage of 1 FTE GP or more. According to our results, underserved areas were mainly found in rural regions. Our findings confirm previous research which concluded that rural areas often suffer from a lack of primary care [29,30]. A surplus in the number of FTE GPs was prevalent in areas with the highest numbers of inhabitants. This indicates that urban areas probably compensate the shortage in rural areas.

Unmet healthcare leads to undesirable consequences: patients are forced to travel greater distances to a GP practice and/or experience longer waiting times before they are seen by a physician. Accessibility problems of GP care may lead to higher utilisation of hospital care, which is more specialised and more expensive, without seeing a GP first [31]. Teljeur et al. (2010) reported that a 1% shortage in GP care supply may result in a 2.4% increase in the demand for hospital care [31]. Therefore, governments and healthcare organisations are being stimulated to promote and facilitate local GP care. Primary care that is available locally enables people to control their own health conditions and prevent diseases; eventually, this may lead to a lower demand for healthcare [32]. Moreover, Pierard (2009) concluded that a larger number of GPs was positively correlated with better health outcomes [33]. However, it needs to be mentioned that a higher supply of physicians may also lead to unnecessary healthcare use.

A flexible GP care system, which is responsive to the demand of the population, is essential to overcome the health problems related to an ageing population and an increase in chronic diseases (National Health Reform, Commonwealth of Australia, 2011). The decision tool presented here is a powerful tool to make both GP care and other primary care disciplines more responsive to the demand of the population. At present, healthcare planners usually base their interventions on national or regional data. The micro level is often overlooked, simply due to a lack of data. Our decision tool can expose geographical differences in the demand for and the supply of primary care; thus, our tool provides health planners with information for the design and implementation of their interventions, like the geographical position of a general practice or a disease specific health plan for a local area. The decision tool also exposes local areas with an expected oversupply or undersupply of healthcare providers. The tool is freely accessible on the Internet and provides demand estimates for GP care, chronic disease care, physiotherapy, dietetics, psychological care, pharmaceutical care and midwifery care. It also provides supply figures for GPs, physiotherapists and midwiferies. Users can select different areas for which they search information and they are also able to download reports. The tool has an average of 2,000 visitors a month. Most users work for regional facility organisations for primary care, local governments, healthcare centres or insurance companies. The usefulness of the decision tool is influenced by the validation of the model. The constructed model could explain almost 13 % of the variance in GP consultation time. It should be noted that the dependent variable was only specified by predictors that are available at a local level for every postcode area in the Netherlands and are updated regularly by *Statistics Netherlands*. The construction as well as the validation of the model is thus restricted by the availability of local predictors. The explained variance could be increased if, for example, information about level of education and lifestyle factors is gathered at a local level and added to the model. Despite the absence of these predictors, the level of explained variance for the number of GP contact minutes can be regarded as acceptable. A previous version of the decision tool has been validated externally using local health survey data from the city of Utrecht from 2003–2006. The study concluded that the Pearson correlation between the two datasets on GP contact was 0.68 [34]. This is a reasonable degree of conformity, especially considering the fact that previous research concluded that the two methods could lead to substantial differences [35].

In our study, the analysis of the geographical differences in the demand for GP care is based on estimated rather than real data, because GP registration data are only available for a small sample of the postcode areas in the Netherlands. In our method, the local demand was estimated based on the composition of the population. So, differences in the estimated demand for GP care between areas could only be explained by the population demographics and the urbanisation of the area and not by GP supply, such as availability and accessibility of GP practices or quality of services. This may be seen as an advantage, because actual healthcare use is influenced by health supply issues. For instance, a large number of GPs in the area may induce healthcare use.

Moreover, not only supply issues may influence actual healthcare utilisation but also different barriers for subgroups in the population to access healthcare such as financial or geographical issues. However, the GP registration data used in this study reflects the national average for healthcare demand for those different subgroups. Still, when interpreting the results of the decision tool, users should always take into account both the local context and their own experience. The decision tool must be seen as a starting point for analysing supply and demand in a region. Additional data should be added to analyse the situation more deeply.

The level of analysis for the present study was postcode level. The classification in postcode areas has been chosen because the supply rates for GPs could only be obtained at postcode level and patients could only be linked to the area characteristics using the postcode of the area. In addition, a study by Reijneveld et al. (2000) showed that there was hardly any difference between the health requirements in deprived and non-deprived areas, regardless of the geographical classification used [36]. In short, we do not believe that the use of postcode level has had a substantial influence on our results.

To decrease the influence of border-crossing to visit a GP, the demand and supply figures of the postcode areas within 3 km of the practice were included in the analysis. However, the distance of 3 km may be considered arbitrary, especially as there are substantial differences between rural and urban areas in the distances between residents and their healthcare providers [37]. In the next update of the decision tool, different distances will be used for urban and rural areas in the analysis of the surrounding areas.

Our study also has some clear strong points. No self-reported data about GP contact were used in the analysis as these may bias the number of visits to the GP. Moreover, the level of analysis of the present study was at micro level. Other studies often analyse at regional or even at national level and extrapolate the means to lower geographical levels, thus neglecting local differences. Furthermore, the method of our study makes it possible to estimate future ratings for the demand for GP care (results not shown in this article). For this reason, the sociodemographic profile of the postcode areas was compiled using predicted figures.

Another clear strong point of the constructed decision tool is the way in which data about primary care are combined, analysed, enriched and made freely accessible. This makes it possible to have an informed discussion about primary care workforce planning in the Netherlands. Moreover, in other countries where local health data are not readily available, the method of the decision tool can also be used. National health and census data should be available and the assumed average workload must be adapted to the country in question.

For further improvements to the constructed decision tool, research needs to be conducted into the factors that could explain the differences between estimated and actual GP contact. Possible explanations may be found at the individual level of patients, the individual level of the healthcare provider, but also at the organisational level of the practice or even in the infrastructure of the practice area; a lack of public transport and/or safe pedestrian walkways may influence access to the GP practice for elderly people. Moreover, to cope with the differences between estimated and actual GP contact, the variable 'perceived health of the population’ could be used as a measure of the need for healthcare. Adding this measure to the decision tool in the future may give more insight into accessibility and availability issues regarding healthcare. Furthermore, plans have been made to integrate other models of healthcare services into the decision tool, such as elderly care and the shift from secondary care to primary care. Also, the statistical analysis can be improved by using a hierarchical regression model, a count model and only using local variables to predict the local demand for care. In the future, we are able to use a more sound statistical model because we are then in the possession of a larger dataset with more respondents per postcode area. Despite the fact that our statistical method can be improved in the future, we do not believe that our method resulted in unreliable outcomes, as the validation study did show [34].

Finally, further research should be undertaken into the implementation of the decision tool and its effect on the way GP care and other primary care disciplines have been organised and whether the amount of underserved areas have diminished as a result.

## Conclusions

This study addresses the growing academic and governmental interest in the appropriate provision of healthcare services to the population of local areas. The constructed decision tool can make a considerable contribution to a primary care system which provides care when and where people need it.

For the results in the other disciplines, the reader is referred to http://www.nivel.nl/vaam (a website in Dutch) or to the report with an extensive description of the method used [38,39],

## Competing interests

The authors declare that they have no competing interests.

## Authors’ contributions

WAdGR and DHdB contributed to the design of this study. DHdB was responsible for the subject of this study. WAdGR was responsible for the day-to-day management, the statistics and produced the first draft of the manuscript. All authors contributed to the write-up of this study. Both authors read and approved the final manuscript.
